# In vitro transplantation of spermatogonial stem cells isolated from human frozen–thawed testis tissue can induce spermatogenesis under 3-dimensional tissue culture conditions

**DOI:** 10.1186/s40659-019-0223-x

**Published:** 2019-03-27

**Authors:** Mahdi Mohaqiq, Mansoureh Movahedin, Zohreh Mazaheri, Naser Amirjannati

**Affiliations:** 1 Anatomical Sciences Department, Faculty of Medical Sciences, Tarbiat Modares University, Tehran, 14115-331 Iran; 2 Basic Medical Science Research Center, Histogenotech Company, Tehran, Iran; 3 Department of Andrology and Embryology, Reproductive Biotechnology Research Center, Avicenna Research Institute, ACECR, Tehran, Iran; 4 Stem Cell Department, Medical Research Center, Kateb University, Kabul, Afghanistan

**Keywords:** Stem cells, Human, Transplantation, Azoospermia, Tissue culture

## Abstract

**Background:**

Sperm production is one of the most complex biological processes in the body. In vitro production of sperm is one of the most important goals of researches in the field of male infertility treatment, which is very important in male cancer patients treated with gonadotoxic methods and drugs. In this study, we examine the progression of spermatogenesis after transplantation of spermatogonial stem cells under conditions of testicular tissue culture.

**Results:**

Testicular tissue samples from azoospermic patients were obtained and then these were freeze–thawed. Spermatogonial stem cells were isolated by two enzymatic digestion steps and the identification of these cells was confirmed by detecting the *PLZF* protein. These cells, after being labeled with DiI, were transplanted in azoospermia adult mice model. The host testes were placed on agarose gel as tissue culture system. After 8 weeks, histomorphometric, immunohistochemical and molecular studies were performed. The results of histomorphometric studies showed that the mean number of spermatogonial cells, spermatocytes and spermatids in the experimental group was significantly more than the control group (without transplantation) (P < 0.05) and most of the cells responded positively to the detection of DiI. Immunohistochemical studies in host testes fragments in the experimental group express the *PLZF*, *SCP3* and *ACRBP* proteins in spermatogonial cells, spermatocyte and spermatozoa, respectively, which confirmed the human nature of these cells. Also, in molecular studies of *PLZF*, *Tekt1* and *TP1*, the results indicated that the genes were positive in the test group, while not in the control group.

**Conclusion:**

These results suggest that the slow freezing of SSCs can support the induction of spermatogenesis to produce haploid cells under the 3-dimensional testicular tissue culture.

## Background

It was reported in 2010 that 0.4% of young patient with childhood cancer can live for a long time [1]. Repeated radiation and chemotherapy cause permanent infertility due to the elimination of spermatogonial stem cells (SSCs) [2], isolation and preservation of testicular tissue before initiating gonadotoxic treatment of cancer, it is a choice to preserve fertility for those who cannot afford sperm storage [3]. A survey among cancer patients between the ages of 14 and 40 during the diagnosis showed that 51% of them wanted to have children in the future [4]. Cryopreservation of testicular tissue can also be helpful for infertile patients who are sampled for therapeutic or diagnostic treatment [5, 6]. Choice of cryoprotectant and procedures for freezing and thawing is very important for Successful cryopreservation of testis. In a study using a slow-freezing procedure for pre-pubertal human testis tissues, dimethyl sulfoxide (DMSO) was introduced to be the most favorable among the cryoprotectants tested, including ethylene glycol, propanediol, and glycerol, based on the maintenance of its architecture along with the viability of constituent cells including spermatogonia, sertoli cells, and those in the interstitium [7]. Culture systems that reproduce the male reproductive cells are various [8]. In vitro culture systems have at least the permission to manipulate the paracrine environment and also to examine the effect of the role of each growth factor individually on the spermatogenesis process [9]. This issue is even more important in cancer patients who are exposed to chemotherapy and radiotherapy treatments because of the high risk of returning cells to cancer patients prior to treatment [10]. Reports on the potential of testicular tissue culture systems have recently begun to be published. Few studies have reported tissue culture optimization. Yokonishi et al. [11] introduced the system of testicular tissue culture on the agarose gel with report of sperm obtaining. They split the immature mouse testicular tissue into small pieces and place it on the agarose gel under culture conditions. Gohbara et al. [9] also reported the release of round haploid spermatids by culturing of the testicular tissue on the agarose gel. Sato et al. [12] used immature mouse testicular tissue fragments to reach fully functional spermatozoa. They isolated mice germ cell from testicular tissue and labeled with green fluorescent protein (GFP) and transplanted into immature azoospermia testis by in vitro transplantation (IVT) and then under tissue culture conditions. After 6 weeks, they reported a functional and adult sperm extraction that could be used for assisted reproductive technology (ART). In another report, Sato et al. [13] put the immature mouse testis tissue that contains only gonocytes and spermatogonial cells precursor under tissue culture conditions on agarose gel and, that leads to full progression of spermatogenesis. They obtained yielded adult mature sperm that can be fertilized by microinjection. They also placed immature mouse testicular tissue after freezing and thawing under tissue culture conditions and reported complete progress in spermatogenesis. In the present study, SSCs isolated from human frozen–thawed testis biopsy were transplanted by in vitro transplantation (IVT) method to mature azoospermia testis model. Then the host testes were placed on agarose gel under 3-D tissue culture conditions. However, until now, the progress of the spermatogenesis process has not been studied by human SSCs in mouse testis niche.

## Methods

### Animals

For each experimental group, 3–5 NMRI mouse (Naval Medical Research Institute) were used at the age of 4 weeks. These mice are treated with Busulfan 40 mg/kg (Sigma, USA) and after 4 weeks, the azoospermia model is developed [14].

### Human testis sample preparation and slow-freezing protocol

This study is based on 5 biopsy samples taken from different obstructive azoospermic patients [sperm positive in their testicular sperm extraction (TESE)] referred to Gandhi Infertility Clinic Center. After performing clinical procedure and confirming active spermatogenesis, and obtaining verbal informed consent from the patients, surplus testis samples were donated to the research laboratory. All stages of this research were based on the approval of the research ethics committee of Tarbiat Modares University with the registration ID IR.TMU.REC.1394.68.

For the freezing and thawing of testicular tissues, the protocol of the Zeng et al. [15] was used. In this way, the small parts of the testicular tissue were placed in a special freezing medium, Leibovitz-L-15 (Gibco-UK) and then placed in a programmable freezer (Planner Cryo 360. 1/7-UK). Testes biopsies were first scraped with scissors and scrapers, about 0.5 cm, and then inside Leibovitz-L-15 (Gibco-UK) cryovials containing 2% fetal bovine serum (FBS) (Sigma-USA) and 10% DMSO (Sigma-USA). The tissues were incubated at room temperature for 15 min and then placed in a programmable freezer. The testicular tissues were cooled at − 20 °C to − 4 °C at − 2 °C/min. At this stage, seeding was done manually using a penny that was previously placed in liquid nitrogen. The cooling cooled from − 4 °C to − 30 °C at − 0.3 °C/min and then cooled down from − 30 °C to − 130 °C at − 10 °C/min. The vials are immersed in liquid nitrogen and then transferred to the storage chamber and held for 48 h. To melt, the vials are kept at room temperature for 1 min and then placed in a 25 °C bath for 1 min. 1 ml of Leibovitz L-15 medium is added to each vial and transferred to sterilized centrifuge tubes. To remove free radicals, the testes are washed twice with the Leibovitz L-15 medium.

### Isolation, culture and confirmation of identification of spermatogonial stem cells

SSCs were isolated by Mirzapour et al. [16] protocol under two steps of enzymatic digestion performed by trypsin (0.5 mg/ml, Sigma, USA), collagenase (0.5 mg/ml, Sigma, USA) and DNase (0.05 mg/ml, Sigma, USA) enzymes. Because of the small number of SSCs present in TESE biopsy, after digestion, in order to enrich of the SSCs and elimination of other cell such as blood cells, these cells are cultured for 2 weeks in Dulbecco’s minimum essential medium (DMEM) (Gibco, UK) supplemented with 10% (v/v) FBS (Gibco, UK) in an incubator with 32 °C temperature and 5% CO_2_. The identification of isolated and purified SSCs was investigated by tracing the PLZF protein as a stem cell marker [17] in colonies derived from cell suspension.

### Preparation support layer for tissue culture

In particular, 1.5% agarose (Carl Roth, German) solution was prepared and sterilized. Segments with dimensions of 1 mm × 1 mm × 1.5 mm of agarose were arranged by scalp considering sterile condition [11]. They were then placed in a six-well Petri dish containing alpha minimum essential medium (αMEM) (Bio-Ideal, I.R.I) with 10% knockout serum replacement (KSR), 60 ng/ml progesterone (Invitrogen, UK), 30 ng/ml beta-estradiol (Pepro Tech, USA), 20 ng/ml epithelial growth factor (EGF) (Pepro Tech, USA), 10 ng/ml human basic fibroblast growth factor (bFGF) (Pepro Tech, USA), 10 ng/ml human glial cell line-derived neurotropic factor (GDNF) (Pepro Tech, USA), and 10 ng/ml leukemia inhibitory factor (LIF) (Royan, I.R.I). Pieces of recipient testicular tissues were placed gently in the middle of the agarose layer after transplantation to prevent them from floating (Fig. 2). The culture medium was replaced twice a week.

### In vitro transplantation of SSCs to the testes

To detect the transplanted cells and purify them from testicular endogenous cells, the cells cultured with a density of about 80% before transplantation for 5 min exposed to a 2 μg di-alkyl indocarbocyanine (DiI) (Eugene.OR, USA) from a 1 ml preservative solution PBS (Invitrogen, UK) was placed at room temperature and then placed in a dark place for 20 min at 4 °C. After ensuring that the cells were stained under a fluorescent microscope, the cell surface was washed with PBS and then isolated from of petri dish by trypsin enzyme (25%) in 0.1% ethylenediamine tetraacetic acid (EDTA) (Sigma, USA). after 3 times washing in the medium, they were transplanted into the host testis. SSCs were transplanted into the host testes below the stereo microscope then they were cut into small pieces and placed under 3-D tissue culture conditions on the agarose support layer. For IVT of SSCs to exited host testes we are using Sato et al. [13] protocol. In this way, using a glassy needle, entered to efferent ductuli and injected cells to the end of the efferent ductuli and the early of ret of testis (Fig. 2). A 10 μL cell suspension containing 100,000 cells was spread into seminiferous tubules and filled about 40–80% of testis.

### Histomorphometric evaluations of the host testes

A total of 5 sections, spaced equally apart, were selected from successive sections of the each testis. After hematoxylin and eosin (H&E) staining of each section, 10 seminiferous tubules with rounded or close-circle sections randomly selected were used to evaluate the testicular parameters. The number of spermatogonial cells, spermatocyte and spermatid per unit volume were measured in each testis by image-j software and previous studies [18, 19].

### Immunohistochemical studies

The testicular tissue fragments of the experimental groups, in addition to the tracing of DiI, were subjected to immunohistochemistry after tissue processing. To confirm the nature of SSCs, spermatocyte and spermatozoa the *PLZF* protein [17], *SCP3* protein [20] and the *ACRBP* protein [21] were detected, respectively. The procedure of immunocytochemistry was performed according to previous study [22]. Briefly, tissues fixed with 4% paraformaldehyde (Sigma, USA) in PBS were Cryo-embedded in OCT compound (optimal cutting temperature) (Sakura, Japan) and cut into 5 µm-thick sections. Incubation with primary antibodies was applied for overnight at 37°. Then the second antibody was applied for 2 h at room temperature in the dark. Nuclei were counterstained with DAPI. Specimens were observed with a confocal laser microscope (TE 2000, Nikon, Japan). The following antibodies were used as primary antibodies: mouse anti *PLZF* antibody (1:100 Santa Cruz Inc, USA), Rabbit anti *SCP3* antibody (1:400 Abcam, UK), Rabbit anti *ACRBP* antibody (1:300 Abcam, UK). The secondary antibodies used were goat anti mouse IgG and goat ant rabbit igG, conjugated with Alexa 488 (1:200, Bio legend UK).

### Molecular studies using real time PCR

In order to prove the presence of different classes of germ cells and to prove that these cells are not due to endogenous spermatogenesis of the mouse testis, testicular fragments of the experimental groups were studied in the of PLZF, Tekt1 and Tnp1 genes. The human specificity of primers designed to differentiate cells. In order to design the primers used in Real Time PCR, the gene sequences from *PLZF*, *Tekt1* and *Tnp1* were obtained from the NCBI database and the sequence of their exons and introns was determined. Primer design was done using the Primer3 online software. Designed primers are blasted to confirm their accuracy and reproduce only the genes’ mRNA sequences. The sequences of the Real Time PCR primers of *PLZF*, *Tekt1* and *Tnp1* genes are shown in Table 1. Total RNA was extracted from the tissue fragments of the different groups by using RNX-Plus™ (Cinnagen, Iran) according to the manufacturer’s recommendations. RNA concentration was then determined using a UV spectrophotometer (DPI-l, Qiagen, IRI). cDNA was synthesized from 1000 ng DNase-treated RNA sample with a Revert Aid™ first-strand cDNA synthesis kit (Fermentase, Lithuania) using Oligo (dT) primers. PCRs were performed using Master Mix and CYBER Green I (Fluka, Switzerland) in StepOne™ Applied Biosystems. The PCR program started with an initial melting cycle at 94 °C for 4 min to activate the polymerase and followed by 40 cycles of a melting step (20 s at 94 °C), an annealing step (30 s at 57 °C), and an extension step (20 s at 72 °C). After the PCR run was completed, the quality of the reactions was confirmed through melting curve analyses. For each sample, the reference gene (β-actin) and the target gene were amplified in the same run. Comparative cycle threshold (CT) method (2^−∆∆CT^) was used to determine the relative quantification of the target genes normalized to a housekeeping gene (β actin). A validation experiment was performed to verify that target efficiencies and reference were approximately equal.

**Table 1:** List of designed primers for molecular studies

Primer sequence	Accession number	Primer	Cell types
Forward: 5′-GTACCTCTACCTGTGCTATGTG-3′ Reverse: 5′-TGTCATAGTCCTTCCTTCATCTC-3′	NM_001018011	PLZF	Spermatogonia
Forward: 5′-CTGACAAGCAGCGGAACAAC-3′ Reverse: 5′-TCTTGGTCAAGGATGGCCTTT-3′	NM_053285.1	Tekt1	Spermatocyte
Forward: 5′-CAATCGCAATTACCGCTCCC-3′ Reverse: 5′-GGCTCCTCTCTGGCTTTGAT-3′	NM_003284.3	Tnp1	Spermatozoa
Forward: 5′-TCCCTGGAGAAGAGCTACG-3′ Reverse: 5′-GTAGTTTCGTGGATGCCACA-3′	NM_001101	β-actin	Internal Control

### Data analysis

All quantitative data in this study were presented as mean ± standard deviation. One-way ANOVA, T-test and Tukey test were used for statistical analysis. The significance level was considered to be P < 0.05.

## Results

**Fig. 1:**
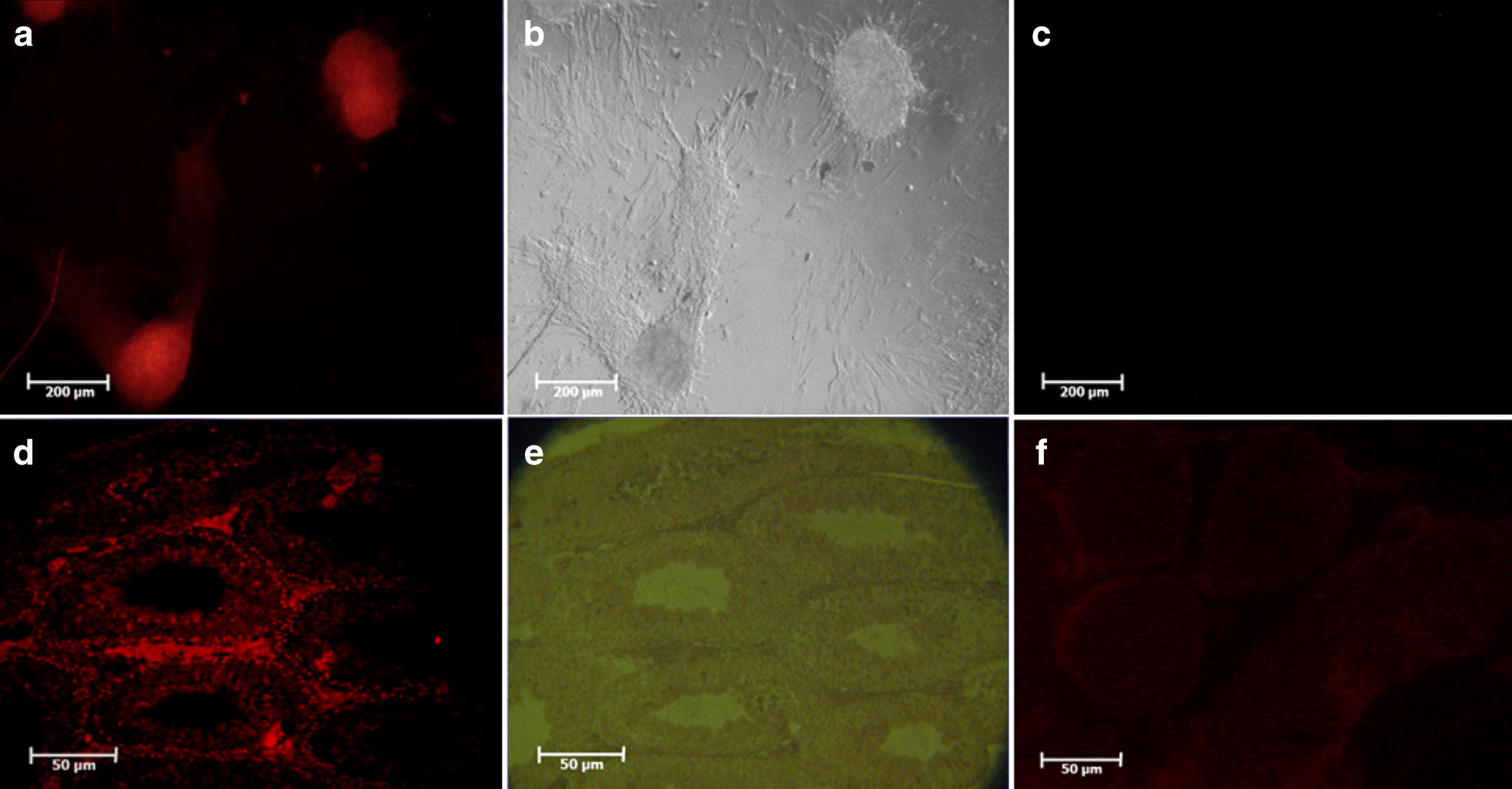
Confirmation of the identification of SSCs. The expression of PLZF protein in colonies obtained from culture of SCs (**a**) and SSCs on seminiferous tubules as positive control (**d**) phase contrast image (**b**, **e**), negative control group without primary antibody (**c**, **f**)

**Fig. 2:**
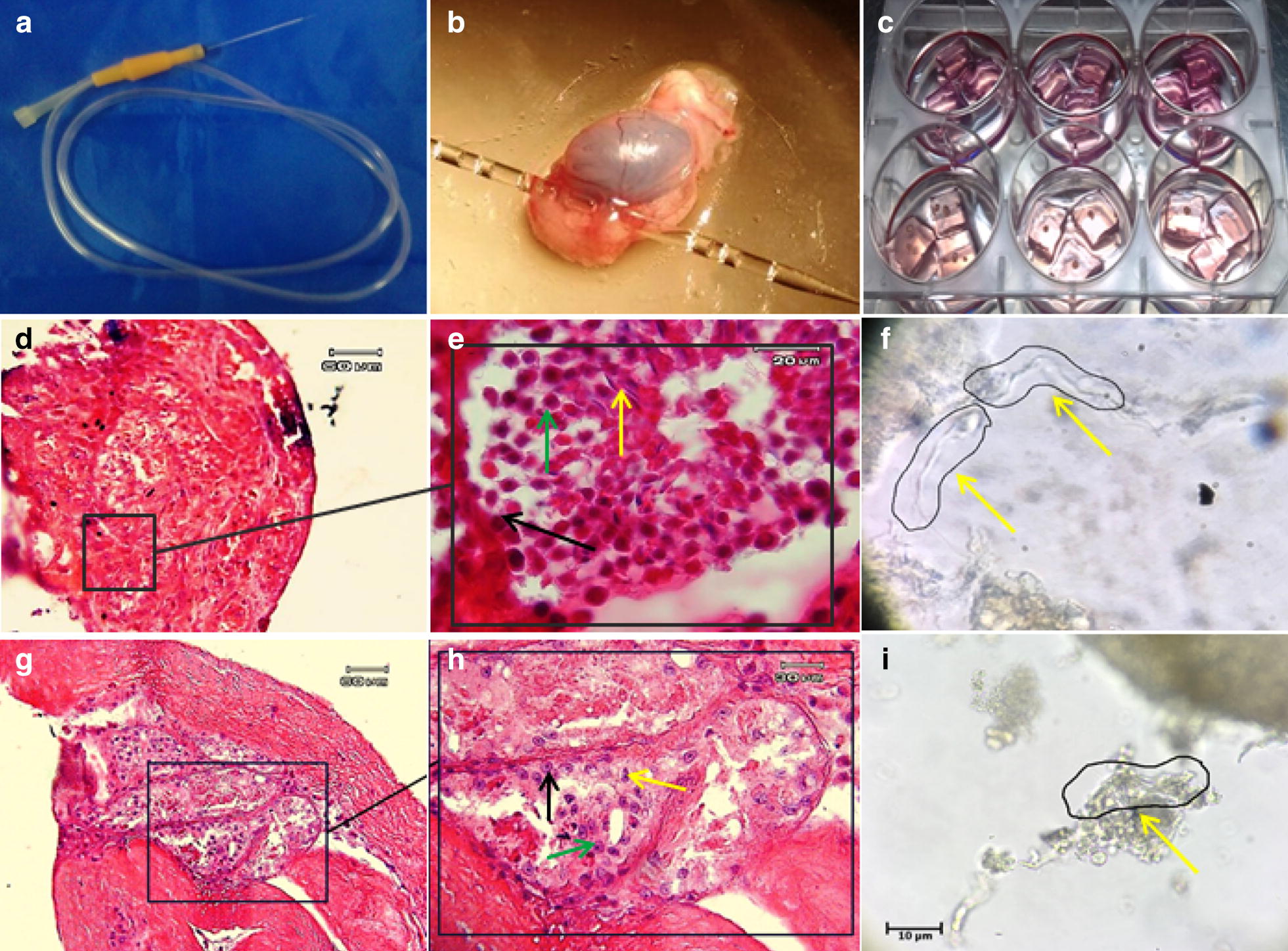
Transplantation of SSCs to host testes and following in organ culture results. IVT of SSs to host testis and organ culture (**a**–**c**). H&E staining of tissue sections IVT group (**d**, **e**) and control group (**g**, **h**). Dynamic dissection of testis fragments after 8 weeks in IVT group (**f**) and control group (**i**). Black arrow: SCs, green arrow: spermatocyte and yellow arrow: long spermatid or sperm like cells

**Fig. 3:**
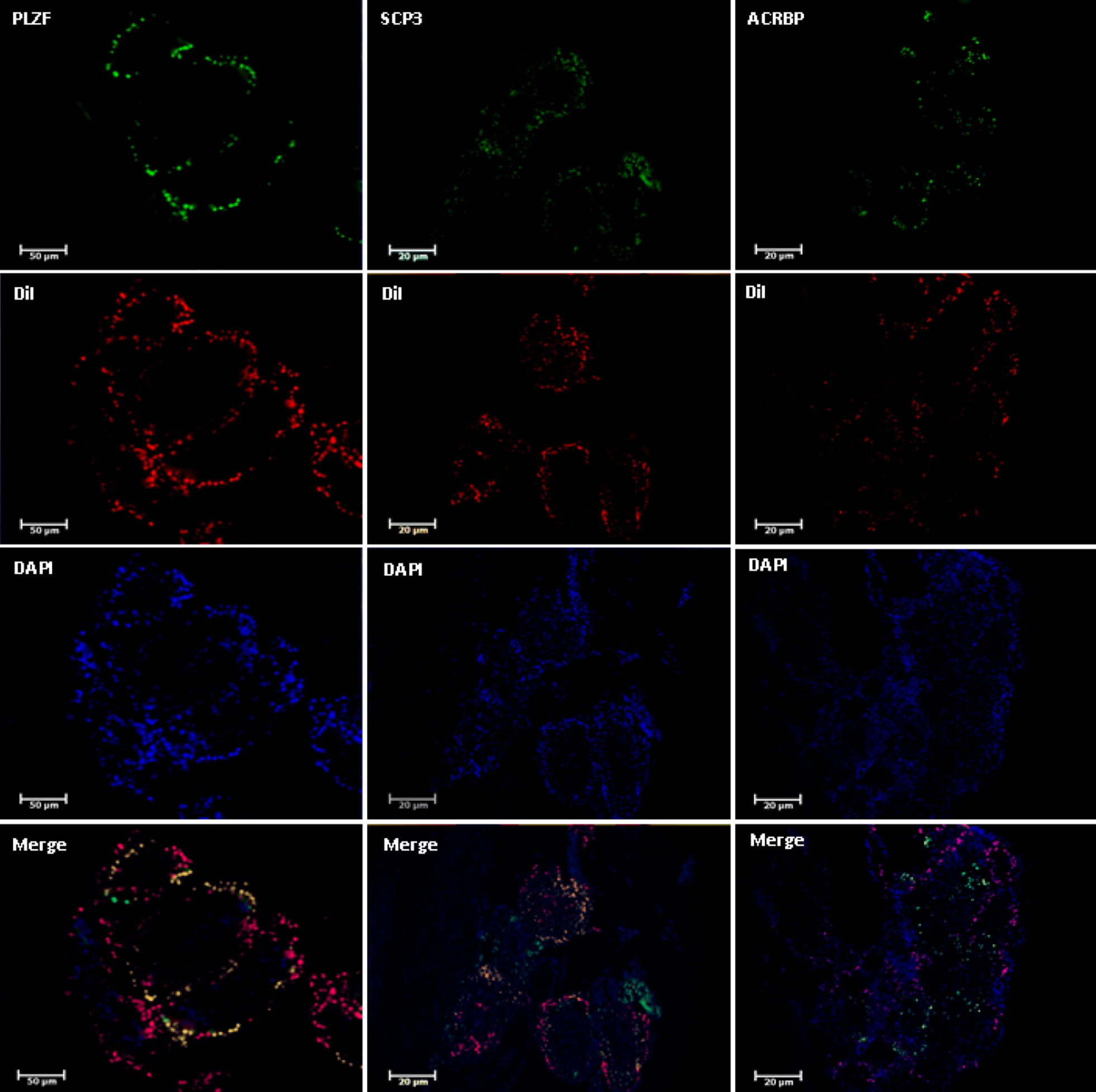
Immunohistochemistry of host testes after transplantation and organ culture. Expression of specific proteins of spermatogonial cells (PLZF), spermatocytes (SCP3) and spermatozoa (ACRBP) and detection of DiI in host testes after 8 weeks of tissue culture

**Chart 1:**
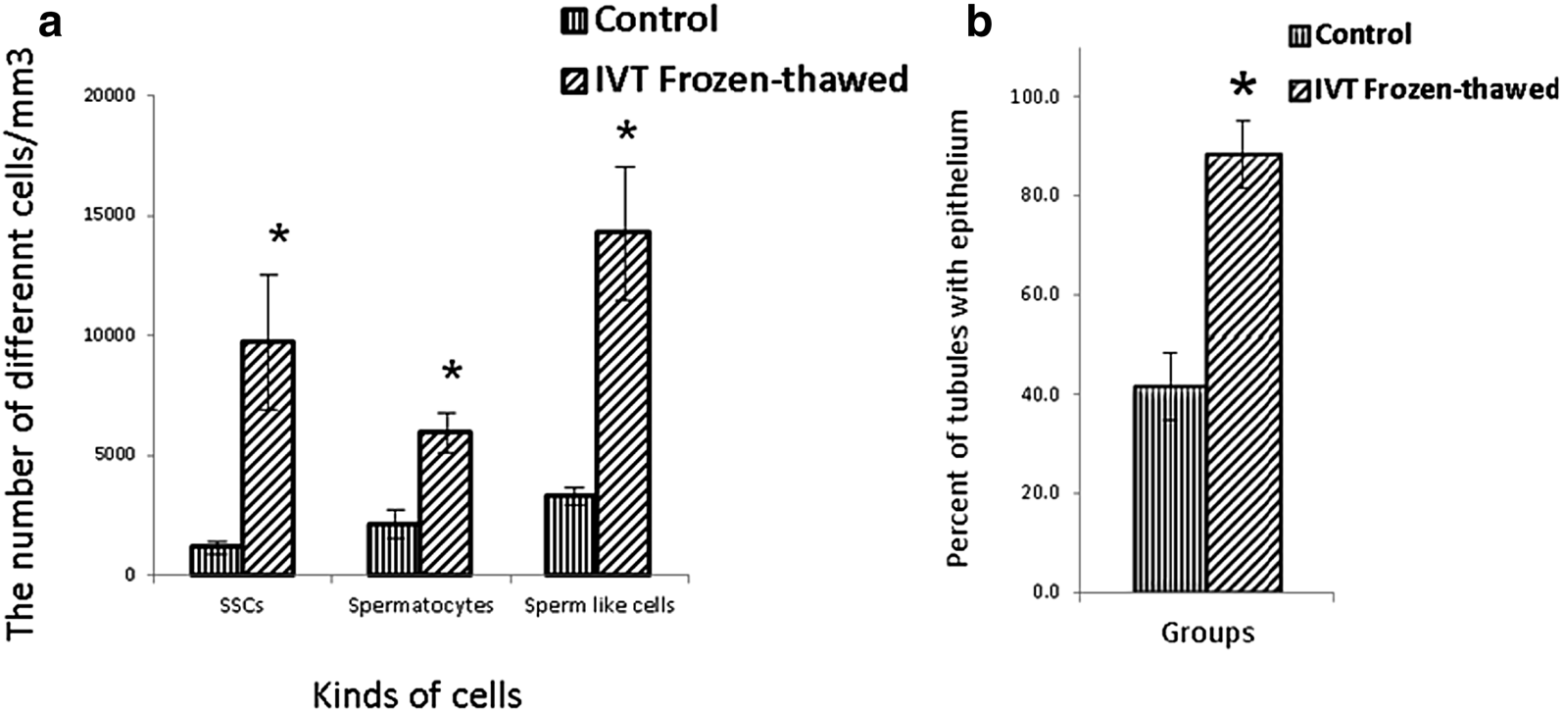
Results of histomorphometric studies in host testes. Number of different types of cells of seminiferous tubule epithelium (**a**) and percentage of seminiferous tubules containing epithelium (**b**) after 8 weeks of tissue culture in different groups. *Significant different with control group (P < 0.05)

**Chart 2:**
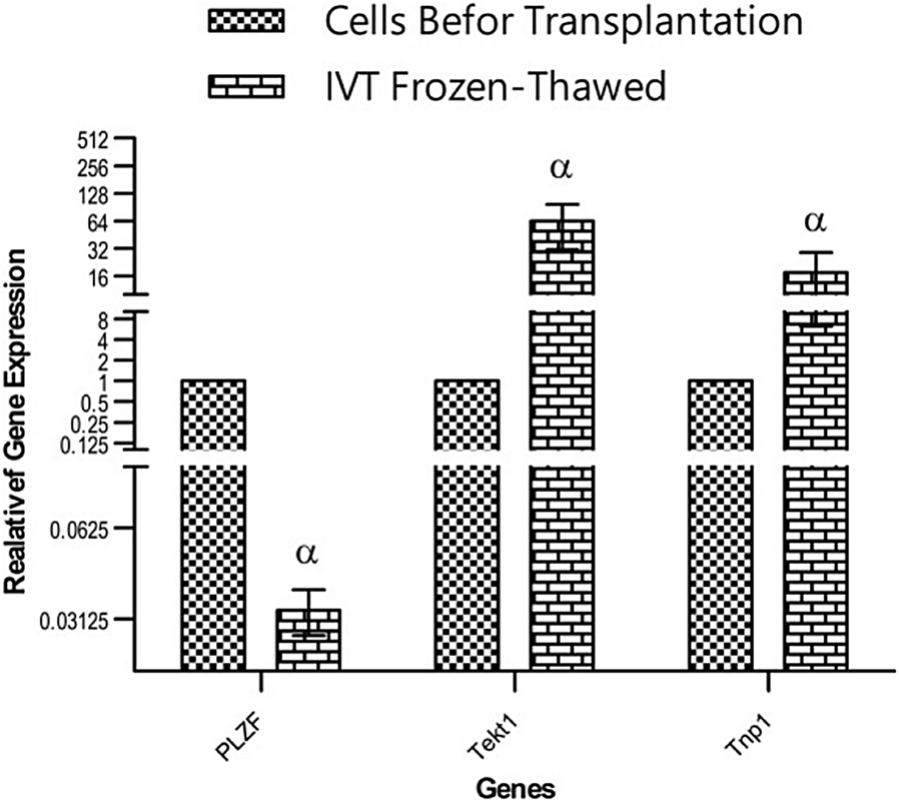
The relative expression of human specific SSCs gene in the host testes after 8 weeks of tissue culture. ^α^Significant different with other group in same gene (P < 0.05)

The identification of the isolated SSCs was confirmed by tracing the *PLZF* protein in the colonies derived from the culture cell suspensions obtained (Fig. 1). In the first and second weeks, the transplanted cells go to the basal area of the seminiferous tubules and place on the base membrane. These cells communicate with sertoli cells and begin to colonize and also differentiate to spermatocyte cell lines. The results of histological studies 8 weeks after transplantation showed a progression of spermatogenesis and relative repaired of epithelium of seminiferous tubules, while most of the testis sections in the control group had no epithelium or poor repaired (Fig. 2). The recovered epithelium contained SSCs that were subsided on the basement membrane as well as spermatocyte cells that gradually moved away from the basement membrane toward the lumen. These cells express the SCP3 protein (Fig. 3) that shows the meiosis divisions and differentiate into sperm cells that are at the end of the epithelium. Also, the histological staining results showed that a series of cells with stretched and dens head were placed in the lumen, which are probably sperm-like cells. This claim was proven by checking the ACRBP protein in these cells. Eventually the repaired epithelium cells express positively *PLZF*, *SCP3* and *ACRBP* proteins in the immunohistochemical studies, which are the specific proteins of SSCs, spermatocytes and spermatozoa, respectively (Fig. 3). Also, most of these cells were DiI-positive, which indicates the human origin of the cells present in the epithelium because we were stained just transplanted cell by DiI in first day before of transplantation. Also the results of dynamic dissection of host testis were shown that sperm like cell were produce after 8 weeks in IVT group but in control group were not (Fig. 3). However, the results of the morphometric studies were shown there were some germ cells in the control group but the number of SCs, spermatocytes and long spermatids, or sperm cells, as well as the percentage of seminiferous tubules with epithelium in the transplant group was significantly higher than the control group without transplantation (P < 0.05) (Chart 1). Transcriptional activity of SSCs, spermatid and sperm like cells which differentiated in vitro were assessed by measuring the relative amount of mRNA encoding for the *PLZF*, *Tekt1* and *Tnp1*, respectively. Due to the fact that primers were designed humanly, no mRNA of germ cells was found in the control group that hadn’t under human cells transplantation. However, the *PLZF*, *Tekt1* and *Tnp1* mRNA were measured positively in transplantation group (Chart 2). The expression of *PLZF* gene in transplantation group was decrease significantly compared to 0th day cell suspension (P < 0.05). However, the expression of Tekt1 and Tnp1 genes in transplantation group was increase significantly compared to 0th day cell suspension (P < 0.05). The expression of Tnp1 in postmeiotic cells in host testis was very important because it shows human SSCs can subsided on mouse testis niche and initiate spermatogenesis to produce haploid germ cells as well as spermatids od sperm like cells.

## Discussion

The transplantation of frozen–thawed testicular tissue or SSCs of cancer patients after the completion of the cancer gonadotoxic treatment period can lead to the re-emergence of malignant cells [4]. In an animal study, it became clear that at least 20 leukemia cells were enough to produce leukemia in the rat model after the transplantation of these cells to host seminiferous tubules [23]. Cell-sorting methods such as FACS can be a way to prevent returning malignant cells, but this is not really true and trustworthy [24]. Xeno-transplantation or xeno-grafting would circumvent the re-emergence of malignant cells, but this method also has its own problems and concerns. One of the concerns is that the patient’s sperm is produced in the body of an animal that is suspected to have a substance, virus, or animal-derived DNA fragments [25]. Xenotransplant of SSCs and testis organ culture in vitro does not carry this risk; however, the ethical and safety implications of this procedure for human clinical samples should be taken into consideration [15].

The transplantation of SSCs into the azoospermia testis model has been carried out by various researchers on various animal models [10]. In the present study, this transplantation was in vitro and then placed under 3-D tissue culture conditions. The results of histological studies and the detection of DiI in the host testis 2 weeks after transplantation indicate the placement of transplanted SSCs on the basic membrane of the seminiferous tubules and the progression of spermatogenesis to the cells in the pulled in lumen centers. Advancing the meiosis in the mammalian spermatogenesis process in a laboratory condition is a tough challenge [26]. A major problem in studying SSCs homing is that it is difficult to track SSCs immediately after transplantation. This is because the concentration of SSCs is very low in the testes cell suspension, and no SSC-specific markers have been identified [27]. The probability that these cells are located in the center of the seminiferous tubules with the nature of haploid or long spermatid sperm cells are confirmed by the results of Immunohistochemical and molecular studies. These findings are consistent with the results reported by Sato et al. [12]. They were done IVT of mouse SSCs into immature azoospermic mice testis that these transplanted cells sit down on a membrane of seminiferous tubules after 7–14 days. Their marking was to track Acrosin GFP in transplanted cells. They reported that after 40–50 days spermatids or sperm cells appeared. However, the exact mechanism of the alignment of transplanted cells on the membrane has not yet been completely transparent [28] but it seems that the way these cells are differentiated after placement on the membrane, into the spermatocyte and spermatid cells is similar to the conditions in the body. Sato et al. [13] conducted another study in which germ cell cells were transplanted into seminiferous tubules in in vitro condition. After placement on the membrane of the seminiferous tubules, these cells began to mitotic and then differentiated into higher-grade germ cells and eventually reached spermatid and haploid sperm cells, which were fertile, and by microinjection, fertile ability the ovum. SSCs transplanted in testes, which were then separated into small pieces and cultured on agarose, migrated towards the basal membrane of seminiferous tubule and settled on it, as in the in vivo process. The efficiency of this homing of SSCs cells appeared surprisingly high and almost comparable to that in vivo. Furthermore, the settled SSCs cells started proliferation over the basement membrane to form colonies, which was also the same as in vivo. The SSCs cells then began to differentiate to form sperm in the cultured testis tissues [13]. Although Mohaqiq et al. [29] have shown that human frozen–thawed SSCs could have successful homing on host testes seminiferous tubules, the mechanism of SSC migration from the lumen to the basal compartment of the seminiferous tubule is not fully understood. The organ culture system would be useful to elucidate SSCs’ behavior, including homing and colonization, in the seminiferous tubules [30]. It seems that the testis tissue culture system can induce the ability to support and resuscitate the spermatogenesis process by preserving the testicular paracrine environment including interstitial tissue and supportive cells in seminiferous tubules. Staub et al. [26] investigated whether the whole process of meiosis in spermatogenic cells could be performed in vitro? Seminiferous tubules fragments from 20 to 28-day-old rats were placed in medium contain FBS and cultured for 4 weeks. They followed differentiation of meiotic germinal cell by four criteria: (i) ultramicroscopic assessment of the different types of germ cells present in seminiferous tubules; (ii) determination of the changes in DNA content per nucleus of the cell population; (iii) assessment of the ability of germinal cells to transcribe genes expressed after completion of meiosis; and (iv) monitoring the fate of BrdU-labeled leptotene spermatocytes. Taken together their results indicate that the whole meiotic process from leptotene spermatocyte to round spermatid can indeed occur in vitro under the presented culture conditions.

## Conclusion

The results of this study indicate that this freezing setup hasn’t major problem to support the progression and induction of spermatogenesis to obtaining haploid cells under IVT system and testicular tissue culture. However, the process of freezing testicular tissue, causing damage to the cells, is undeniable. But this method for cancer patients can only be a way of maintaining fertility. Therefore, the methods of testicular tissue freezing should be optimized to minimal damage possible.

## Abbreviations

ART: assisted reproductive technology
DMEM: Dulbecco’s minimum essential medium
DMSO: dimethyl sulfoxide
FBS: fetal bovine serum
GFP: green fluorescent protein
H&E: hematoxylin and eosin
IVT: in vitro transplantation
KSR: knockout serum replacement
SSCs: spermatogonial stem cells
αMEM: alpha minimum essential medium
